# Groundbreaking Research from NIDA Addressing the Challenges of the Opioid Epidemic

**DOI:** 10.1007/s13181-024-01041-w

**Published:** 2024-11-08

**Authors:** Nora Volkow, Leslie R. Dye

**Affiliations:** 1 Director of the National Institute On Drug Abuse at the National Institutes of Health, Three White Flint North, 11601 Landsdown Street, North Bethesda, MD 20852 USA; 2 OneFifteen, 257 Hopeland Street, Dayton, OH 45417 USA

**Keywords:** Opioids, Overdose, Naloxone, Fentanyl, Justice system

## Abstract

The 2024 ACMT Ward and Ryan Donovan Memorial Fund lecture was presented by Dr. Nora Volkow, the director of the National Institute on Drug Abuse (NIDA) at the National Institutes of Health (NIH). This article in an edited version of her keynote address during ACMT’s 2024 Annual Scientific Meeting. During the course of her talk, Dr. Volkow discussed the historical factors contributing to the ongoing global opioid epidemic, examined the evidence behind different front-line and policy strategies used to battle the opioid epidemic, and highlighted the importance of recent cultural changes that support more deliberate screening for substance use disorders and pathways for initiating treatment of opioid use disorders in vulnerable populations. An urgent need exists to improve inclusion of social and structural determinants of health in implementation science addressing opioid use disorders, with better attention to special populations, including Native American Indians and Alaskan Natives, African Americans, those over 65 years of age, and teenagers.

## Background of the Donovan Lecture

With the passing of the American College of Medical Toxicology (ACMT) past president and Medical Toxicology Foundation board member Ward Donovan, MD, FACMT, the Medical Toxicology Foundation is the beneficiary of a legacy gift from his estate. This gift combined with the Ryan Donovan Memorial Fund has been used to establish the Ward and Ryan Donovan Memorial Fund. Since 2022, this fund has been used to support a visiting lectureship and/or panel discussion annually at the ACMT Annual Scientific Meeting (ASM) to provide education on the topic of opioid and other substance use disorders, with the goal of increasing the awareness, ability, and opportunity for medical toxicologists to increase their understanding and practice in this area.

**Fig. 1:**
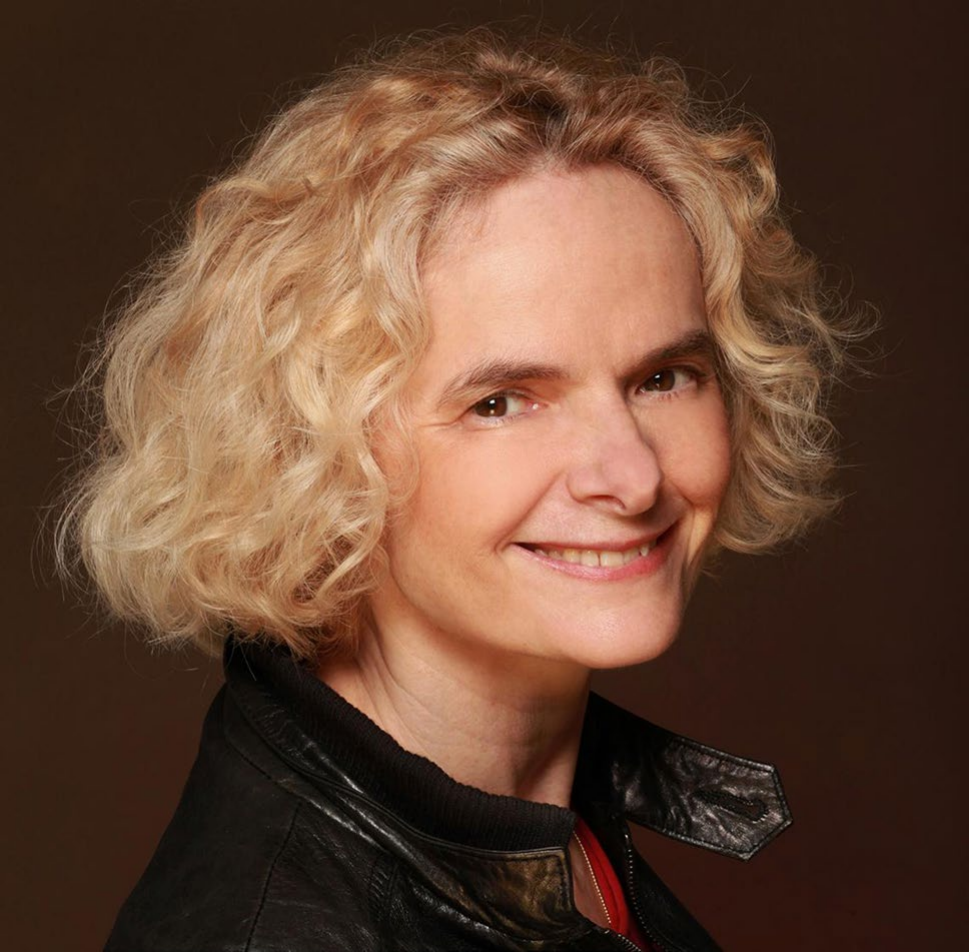
Dr. Nora Volkow (*photo courtesy of Mary Noble Ours*)

At the 2024 ACMT Annual Scientific Meeting, the ACMT Addiction Toxicology Committee was honored to have Nora Volkow, MD, the Director of the National Institute on Drug Abuse (NIDA), accept the invitation to be the ASM Keynote and Ward and Ryan Donovan Memorial Fund lecturer in Washington, DC (Fig. 1).

## Introduction of Dr. Nora Volkow (Dye)

Nora D. Volkow, M.D., is Director of the National Institute on Drug Abuse (NIDA) at the National Institutes of Health. NIDA is the world’s largest funder of scientific research on the health aspects of drug use and addiction.

Dr. Volkow's work has been instrumental in demonstrating that drug addiction is a brain disorder. As a research psychiatrist, Dr. Volkow pioneered the use of brain imaging to investigate how substance use affects brain functions. Her studies have documented how changes in the dopamine system affect the functions of brain regions involved with reward and self-control in addiction. She has also made important contributions to the neurobiology of obesity, ADHD, and aging.

Dr. Volkow was born in Mexico and earned her medical degree from the National University of Mexico in Mexico City, where she received the Robins Award for *best medical student of her generation*. Her residency in psychiatry was at New York University, where she earned a Laughlin Fellowship from The American College of Psychiatrists as one of *10 outstanding psychiatric residents in the United States.*

Much of her professional career was spent at the Department of Energy’s Brookhaven National Laboratory in Upton, New York, where she held several leadership positions including Director of Nuclear Medicine, Chairman of the Medical Department, and Associate Laboratory Director for Life Sciences. Dr. Volkow was also a professor in the Department of Psychiatry and Associate Dean of the Medical School at The State University of New York at Stony Brook.

Dr. Volkow has published around a thousand peer-reviewed articles, written 113 book chapters, manuscripts, and articles, co-edited *"Neuroscience in the 21st Century,"* and edited four books on neuroscience and brain imaging for mental and substance use disorders.

Dr. Volkow received a Nathan Davis Award for Outstanding Government Service, was a Samuel J. Heyman Service to America Medal (“Sammies”) finalist and is a member of the National Academy of Medicine and the Association of American Physicians. Dr. Volkow received the International Prize from the French Institute of Health and Medical Research for her pioneering work in brain imaging and addiction science; was awarded the Carnegie Prize in Mind and Brain Sciences from Carnegie Mellon University; and was inducted into the Children and Adults with Attention-Deficit/Hyperactivity Disorder (CHADD) Hall of Fame. She was named one of Time magazine's "Top 100 People Who Shape Our World"; one of "20 People to Watch" by Newsweek Magazine; Washingtonian magazine’s "100 Most Powerful Women"; "Innovator of the Year" by U.S. News & World Report; and one of "34 Leaders Who Are Changing Health Care" by Fortune magazine.

She accepted the Emmy for NIDA when they won it with The National Institute on Alcohol Abuse and Alcoholism for HBO’s *The Addiction Project.*

For her scientific contributions and her personal commitment to understanding addiction, in 2018 Georgetown University Medical Center bestowed upon her its highest honor — the ***Cura Personalis*** Award.

*Cura personalis* means “care of the whole person,” and suggests individualized attention to the needs of others, distinct respect for unique circumstances and concerns, and an appropriate appreciation for singular gifts and insights.

## Groundbreaking Research from NIDA Addressing the Challenges of the Opioid Epidemic (Volkow)

### History and Evolution of Our Current Crisis

We, as physicians and many of the clinical providers contributed to this crisis. Aggressive practices were undertaken by the pharmaceutical industry to say that “If you have pain, you are not going to become addicted to opioids. Opioids are quite safe. And even if there are some effects, you become tolerant so you shouldn’t worry about it.”

**Fig. 2:**
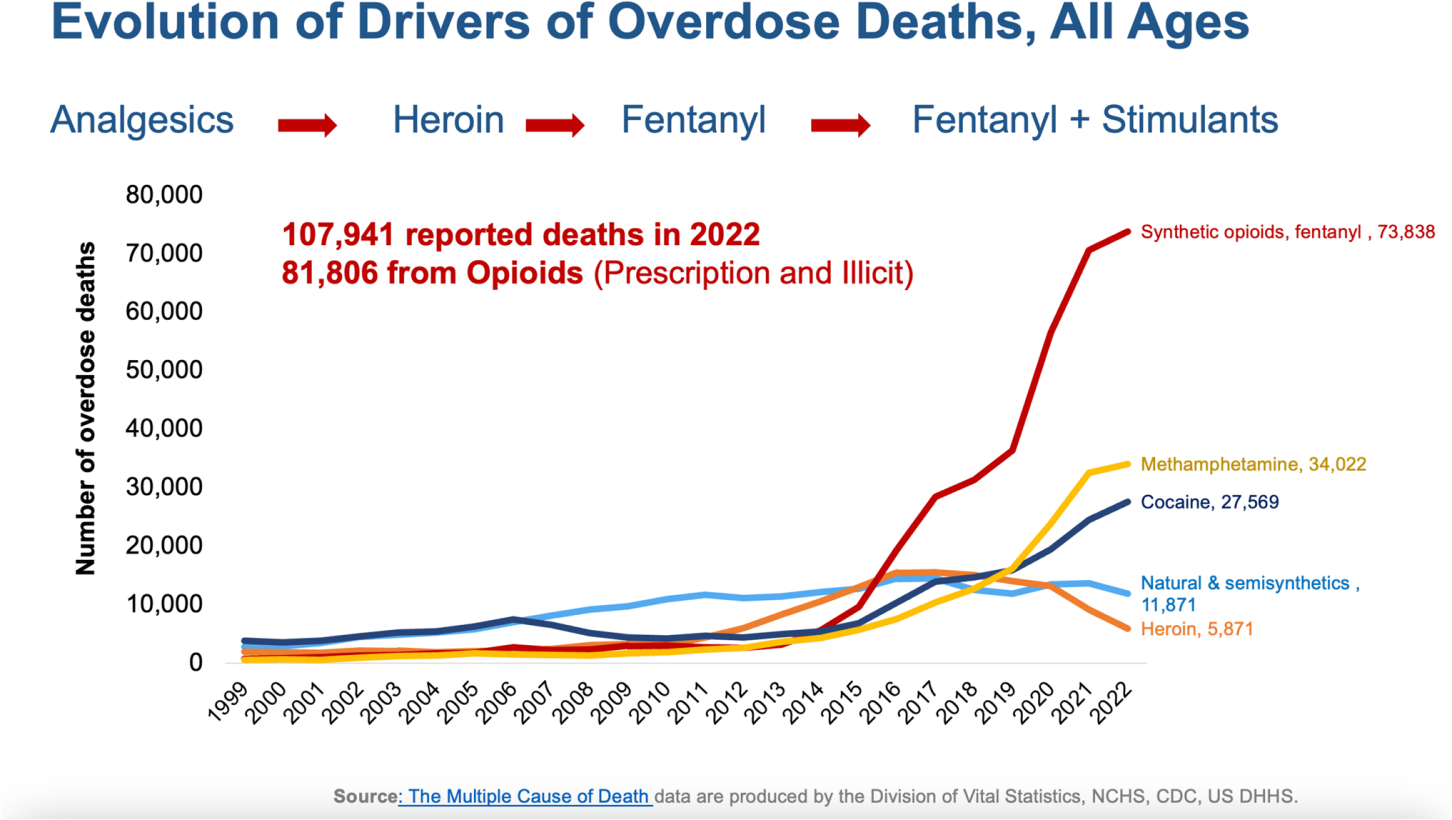
Cause of overdose death based on coroner report in the United States by year. Sky blue line indicates natural and semi-synthetic prescription pain medications

Unfortunately, that belief was utterly wrong, and we did not see evidence of this until multiple agencies started to pay attention in the early 2000’s. I had noticed this trend since 2003 when I took the position of NIDA Director (Fig. 2) [1]. As I was looking at the numbers, I realized that the data did not make sense. What surprised me was that, unlike in the past, teenagers were reporting misuse of prescription opioids. Since this was a new development, I concluded that these were prescriptions written by physicians that were being diverted.

The CDC began following the data and recognized that there was a need for guidelines to avoid overprescribing. After that, heroin use went up as people found it harder to get pills and they were addicted to opioids. Of course, the drug cartels immediately took advantage of that circumstance and something dramatic and very malicious happened. We began to see heroin contaminated with fentanyl.

And why was fentanyl initially added to heroin? It is illicitly manufactured, synthetic, and provides greater profit. You don’t need to cultivate poppies. It has extremely high potency (at least 100 times more potent than morphine or 50 times more potent than heroin) and requires much smaller volumes to produce the same effect.

Innovation led to novel delivery, including through the mail, so heroin stopped being contaminated, but was replaced by fentanyl. Fentanyl was then used as a contaminant with other substances like cocaine. Cocaine is more expensive to cultivate and export than fentanyl. So, you just put a little fentanyl in the cocaine, and you have a very powerful drug. In 2015 and 2016, we started seeing an increase in mortality from methamphetamine. Most of those cases were contaminated with fentanyl.

**Fig. 3:**
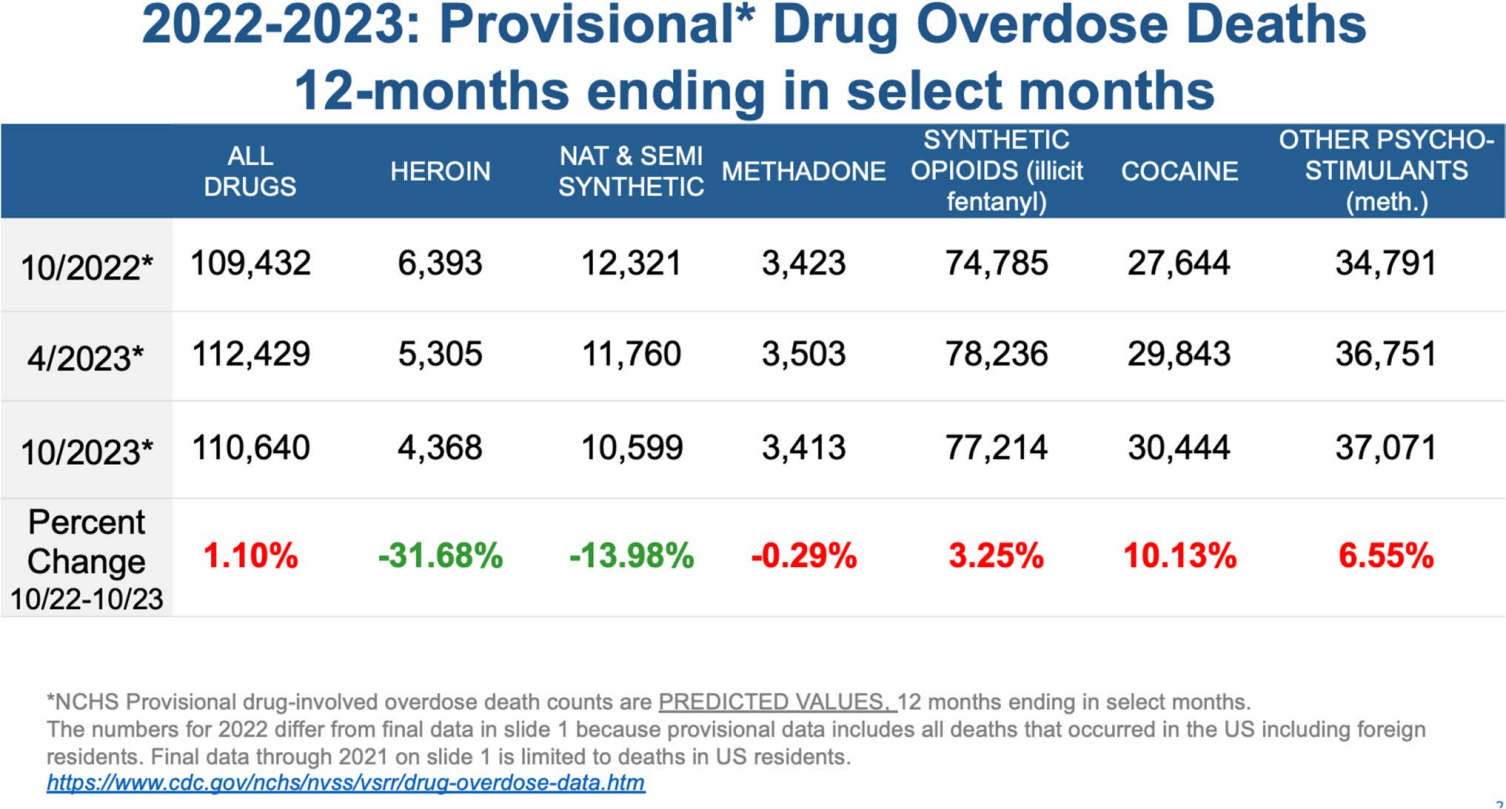
2022–2023: Provisional Drug Overdose Death causes

Today it is fentanyl and fentanyl analogs (Fig. 3). We are starting to see the emergence of nitazines. It is like we are on a treadmill trying to identify the patterns of drugs that are being laced with other adulterants. And we need to be able to detect them faster so we can intervene. If we don’t know what is causing the mortality, we cannot do proper interventions. We know naloxone works for fentanyl, but what about drug combinations?

If we look at the latest numbers that ended in October 2023 (Fig. 3), more than 110,000 died of overdoses. Many of them are quite young. More than 110,000 deaths per year and 100% preventable. I’m not even speaking about all the social disruption that illicit drugs are creating along with crime. Many more deaths among young people during the COVID pandemic were from drug overdoses than from COVID. *This is the reason why I said this is the most urgent crisis that we currently have from the health care perspective. And what we’re seeing is that it continues to increase despite all these aggressive interventions from multiple agencies.*

### Combining Drugs

I do research on brain imaging and am studying the effects of medications in individuals with opioid use disorder. We have completed studies on 66 of them with complex protocols. Not a single one of them had heroin in their urine despite the fact that was what they claimed they had taken. All of them tested positive for fentanyl. These research participants are mostly from Baltimore and other areas in Maryland. Similar reports are described in many other states. So, fentanyl use is what people are basically dying from, whether by itself or with a combination of other drugs.

Depending on what drugs they are combining, you may have very different challenges in reversing the overdoses or even treating them. If you have someone who has taken both methamphetamine and fentanyl of course naloxone may reverse the hypoxemia from fentanyl, but what about arrhythmias triggered by methamphetamine? As a psychiatrist I am very frustrated by having such a limited number of treatments for addiction.

### Ongoing Progress

#### Medications that Assist with SUD Treatment

**Fig. 4:**
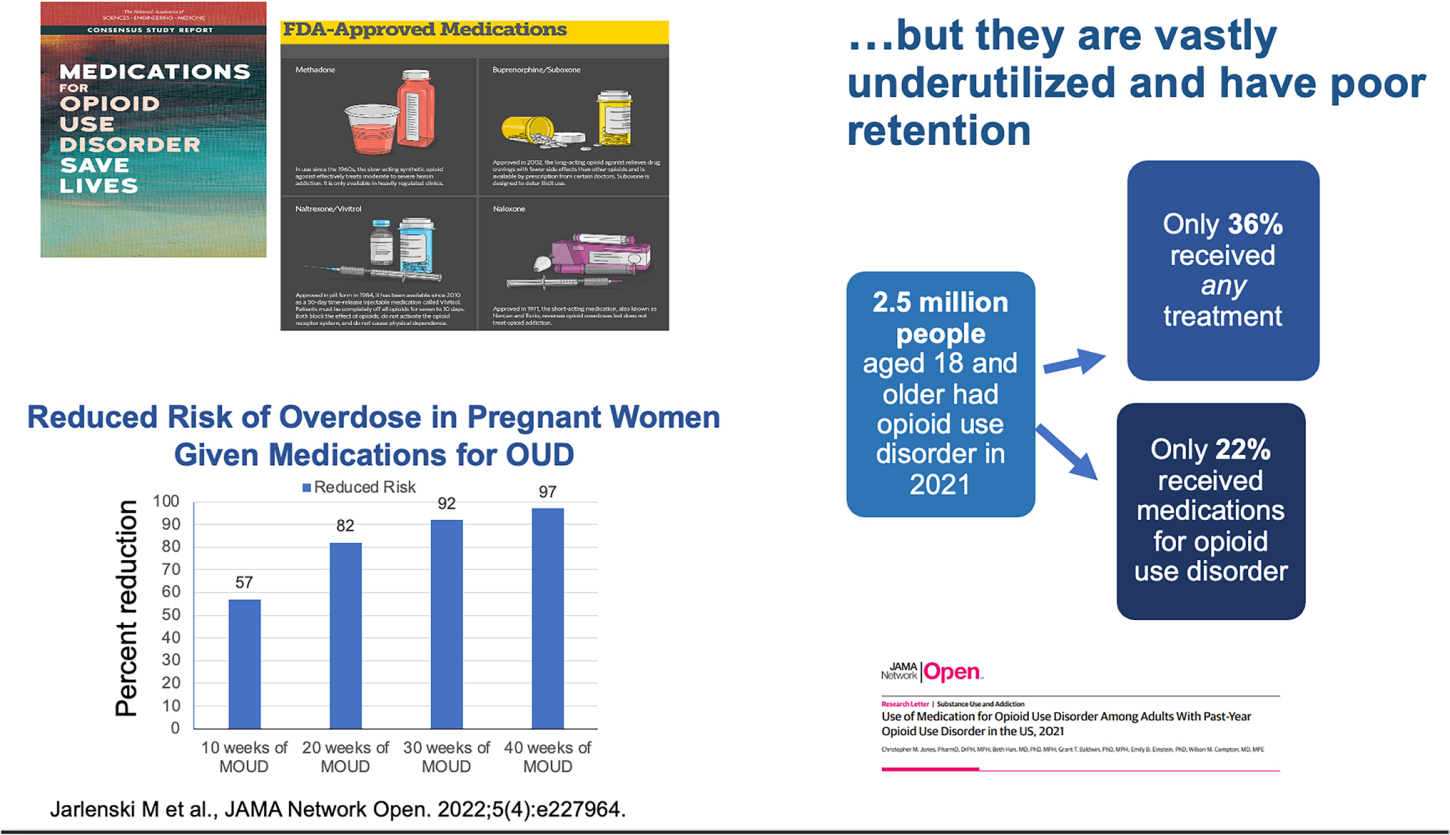
Medications for opioid use disorder

We have three different classes of medication and I’m very grateful to have each of these in our armamentarium for treating opiate use disorders (OUD), but we urgently need more [2]. The ones we currently have are methadone, buprenorphine, and extended-release naltrexone. These medications can protect from overdose. In one study, if pregnant women took their buprenorphine throughout the whole pregnancy for 4 weeks or more, they basically prevented overdoses by 97%. (Fig. 4) [3].

The first challenge is you need to give the medication to the person. And second, you need the person to keep taking it. Of those people that have opioid disorder, perhaps 22% and certainly no more than 30% will take the medication prescribed. Approximately 50% will discontinue by 6 months. (Fig. 4).

#### Reversal Agents

Both naloxone and naltrexone are opioid receptor antagonists that will reverse overdose, if you have the correct dose. Now if you also have a stimulant, that’s a different story. So, these are major challenges. From my perspective, in order to be successful in combatting the drug crisis we need to advance science because otherwise we won’t succeed in controlling or preventing SUD and the morbidity and mortality associated with it. In the meantime, we need to use the tools that we have to protect people from dying and help them achieve recovery.

#### Implementation Science

Implementation science is the specific task of determining if there is a huge gap between scientific findings and real world practices. Multiple studies have reported that there are insufficient addiction treatment clinics to take care of people with SUD and provide medications for OUD. But there is nothing that tells us these medications or the treatment of people with addiction should be done in a separate system, which is the way that we have dealt with treatment of SUD in this country. It is a separate system from the healthcare system.

#### Cultural Change

NIDA has used its Clinical Trials Network (CTN), which is composed of 17 sites across the United States to create new partnerships with health care to embed models of care that allow for providers to screen and treat SUD and to evaluate outcomes. For example, this research has shown that engaging emergency departments in the initiation of treatment for OUD is very effective. It improves patients’ retainment in treatment and diminishes the likelihood that these patients come back to the emergency department with an overdose or other adverse consequences.

We also are implementing a model to evaluate how to optimally do screening and treatment of people with OUD in primary care because this is the first line in healthcare and the most extensive one that we have to reach the highest number of people. Similarly, we are engaging in showing and documenting integration of OUD with HIV care or with hepatitis C (HCV) care or other infectious diseases.

Other work at the Clinical Trials Network has shown that involvement of pharmacies to provide methadone for OUD improves access to treatment instead of having to attend an opioid treatment program (methadone clinic), on a daily basis that for many means having to travel long distances to attend it. The bottom line is, we’re trying to transform the way that the health care system gets involved with the engagement in treatment of patients with OUD.

This requires education and challenging current models of reimbursement which are inadequate. To change the health care system, we must ensure that physicians, nurses, pharmacists, and everyone in health care have expertise in the treatment of addiction and that they are properly remunerated for their work.

#### Justice System

Similarly, another NIDA priority area in research has been to advance SUD treatment in justice settings. This is very different from what we normally do at the NIH where we work with hospitals, clinics and increasingly community providers, but we don't work with prisons or jails. To achieve the goal of expanding treatment to people with an OUD, not only health care but also the justice system provides unique opportunities. Of those who die from overdoses, 25 percent have been in a justice setting in the year prior to their death [4]. If we build effective and sustainable models to do screening and treatment interventions for OUD and other SUD in carceral settings many future overdose deaths will be averted. This will also decrease rates of re-incarceration.

**Fig. 5:**
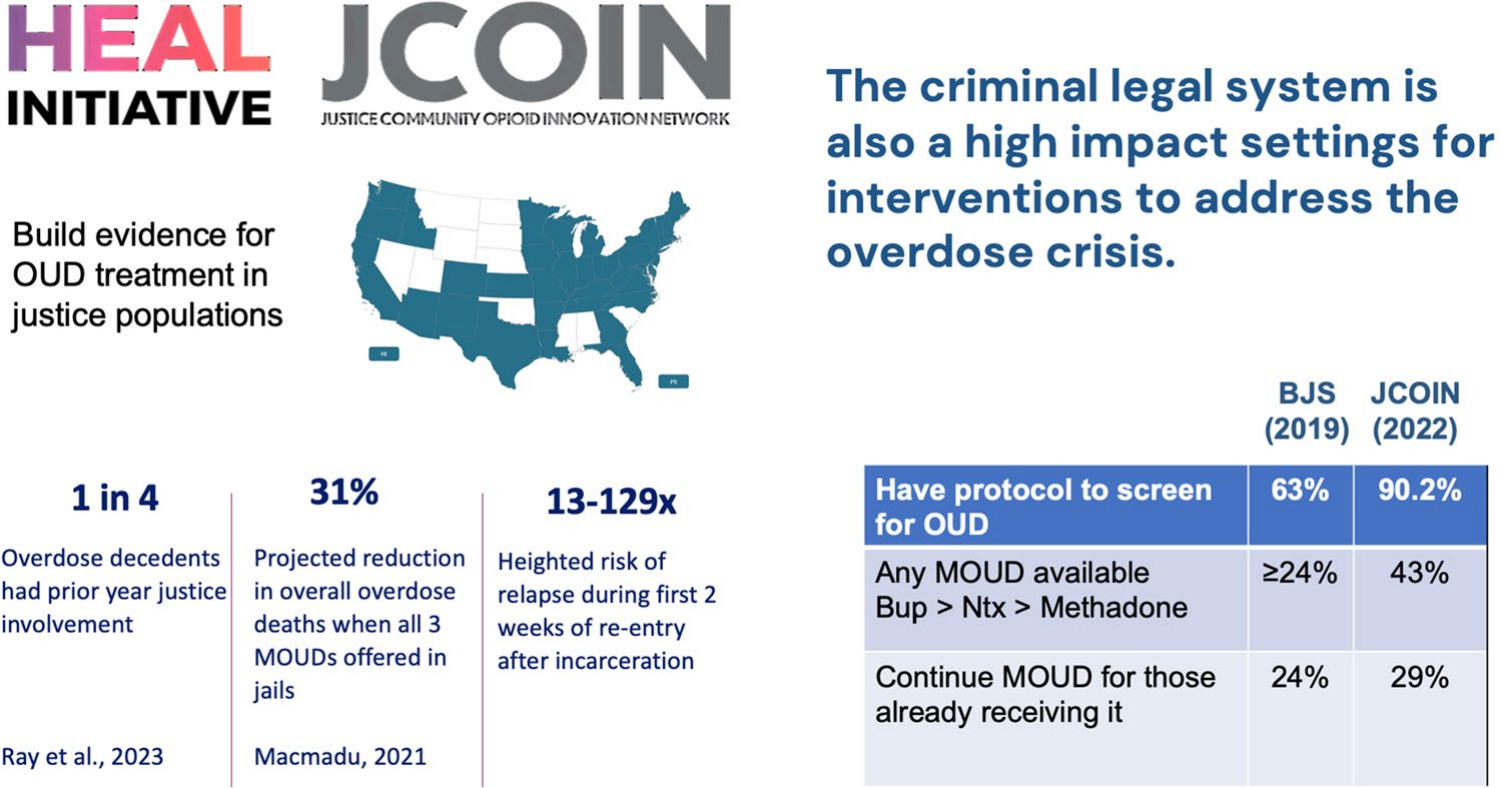
Medication for OUD in the justice system

A demonstration project in Rhode Island that implemented all medications for OUD in prisons and jails showed that they reduced mortality by 70% in this population which translated into a 30% reduction in overdoses for the whole state [5]. How do you change a culture that has been very reluctant? The first step is to conceptualize addiction as a disease that benefits from treatment as opposed to a crime that requires a punitive intervention. The data on averted overdose mortality speaks for itself for it indicates that providing medication for OUD significantly improves the outcomes of people in the justice system (Fig. 5).

Changes in telehealth and how we prescribe OUD medications that were brought about by the COVID pandemic have been made permanent. Some of these facilitated bringing medications to jails and prisons that don’t have doctors. There is a much greater awareness that treatment of individuals in jails or prisons or just before they get released is associated with better outcomes. States are also allowing prisoners to keep their insurance in prison or providing health support when they get released.

### Why Fentanyl is a Game Changer

Fentanyl is extremely potent as an analgesic, but also extremely addictive and it produces physical tolerance very, very rapidly, as well as severe withdrawal upon lowering of blood levels or drug discontinuation. The rate of tolerance development is much faster than for heroin. Most of the fentanyl that is misused is illicitly manufactured and does not have the quality control that exists when manufactured for medical use. When someone buys an illicit drug, they do not know whether it has fentanyl nor the amount it might contain nor what other contaminants it might contain.

There’s another aspect about fentanyl that makes it unique. It’s not just the potency that is high, but it is also highly lipophilic. This is relevant because the drug gets to the brain faster than heroin. This is important because the faster a rewarding drug gets into the brain, the more addictive it is. The other worrisome issue is that fentanyl’s fast brain entry leads to a faster respiratory depression than observed with heroin. That means the window for giving naloxone in order to reverse an overdose is reduced.

Additionally, fentanyl is much more likely than heroin to produce chest rigidity syndrome. Because you cannot breathe, you cannot expand your lungs, which further contributes to the high mortality rates seen with fentanyl.

### Ongoing Research

We are working with researchers to develop sequestering molecules that can trap methamphetamine or fentanyl in the blood and prevent it from continuing to enter the brain or other systems. We’ve also been developing innovation and technologies to monitor for overdose that can automatically set an alarm and initiate a response system including automatic naloxone delivery. Though this automatic delivery system is more likely to be beneficial for people that are taking opioid medications for pain in the future with the input from people who use illicit opioids, we might be able to develop more acceptable prototypes for individuals with OUD. We are also funding research to test new molecules that can directly stimulate respiration and expand our options which are currently based on antagonists of mu opioid receptors.

From genetic and neuroimaging studies, we have come to realize that there are more similarities than there are differences in addictions to different substances. There are genes that make you vulnerable to addiction in general and neural circuitry that is disrupted in people who are addicted, regardless of the type of substance addiction. This knowledge can be used to design molecules (like Dopamine D3 receptor antagonist) or therapeutics such as neuromodulation to target these common neural circuits. Polysubstance misuse is increasingly becoming more prevalent and complex and its treatment more challenging and requires the urgent development not only of new treatments but also expanded screening and access to treatment and support for people with substance use disorders.

### Successes

The approval of nalmafene hydrochloride nasal spray by the FDA is a recent success. That is an opioid receptor antagonist that has the advantage over naloxone that it can get to the brain faster [6].

**Fig. 6:**
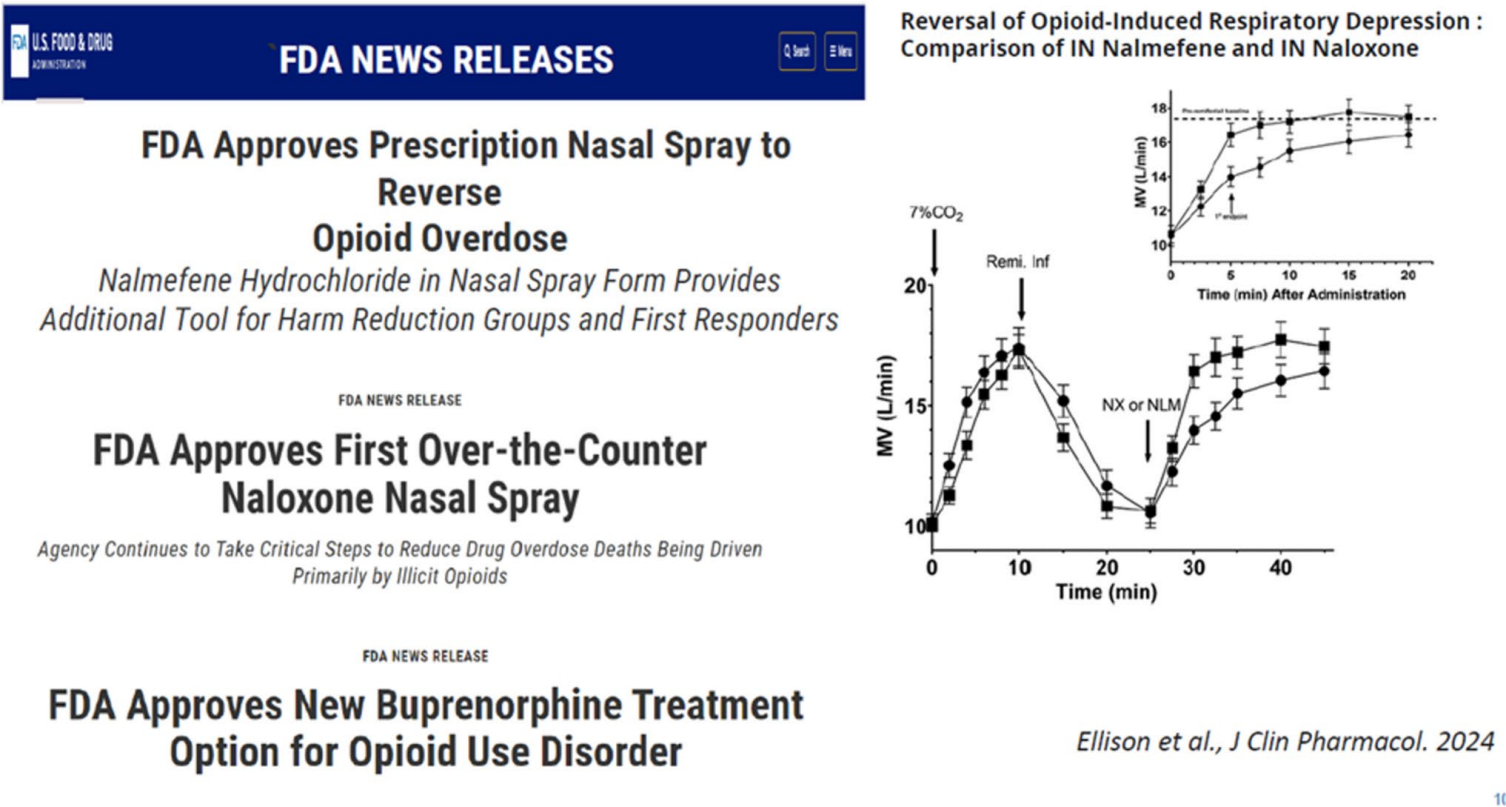
Naloxone, Nalmafene and Injectable Buprenorphine

Having naloxone is a success. The FDA finally approved extended-release buprenorphine treatment. This allows us to give an injection for one week or one month. These are powerful tools that we currently have (Fig. 6). Though the buprenorphine waiver requirement was believed to be a main culprit for the lack of buprenorphine access in the country, now that the waiver requirement has been removed, we have not seen the expected uptick in buprenorphine prescriptions.

### Xylazine

**Fig. 7:**
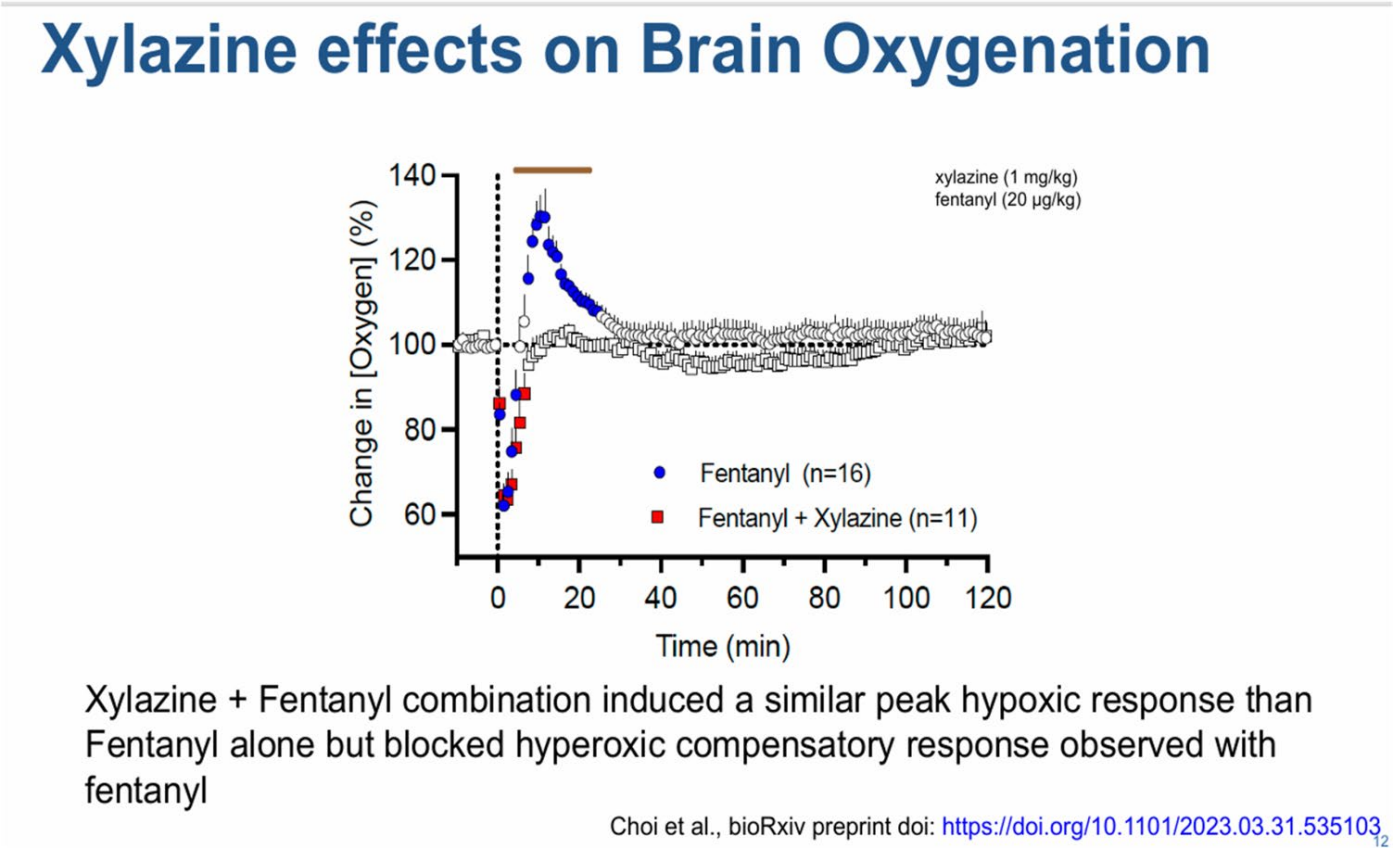
Effects of xylazine mixed with fentanyl on brain oxygenation

In 2019 it was predominantly reported in the northeast that fentanyl was being contaminated with xylazine. Since then, it has proliferated. It is surprising because xylazine is an alpha-adrenergic agonist used as a sedative-hypnotic in veterinary medicine. Xylazine increases the volume of drug being injected but it is also pharmacologically active. Many people do not like the effects. Others do like it because they claim that when added to fentanyl its effects last longer. The effects on respiration and hypoxemia last longer when xylazine is mixed with fentanyl and dramatically reduce the concentration of oxygen in the blood (Fig. 7) [7]. Not only is this relevant for mortality but also to neurotoxicity from repeated bouts of hypoxemia to the brain.

About three years ago, horrible wounds were observed to be developing in fentanyl/xylazine users that were growing extremely rapidly. Physicians started to debride xylazine users’ wounds to try to preserve and clean the tissue. In many cases, that rapidly resulted in amputation. They stopped debriding them. The wounds sometimes were not at the site of injection. If you are giving an alpha agonist, you will be producing vasoconstriction in the peripheral system. How does this ultimately affect the risk of mortality? In principle people have believed it is probably not good.

### Wastewater Testing

**Fig. 8:**
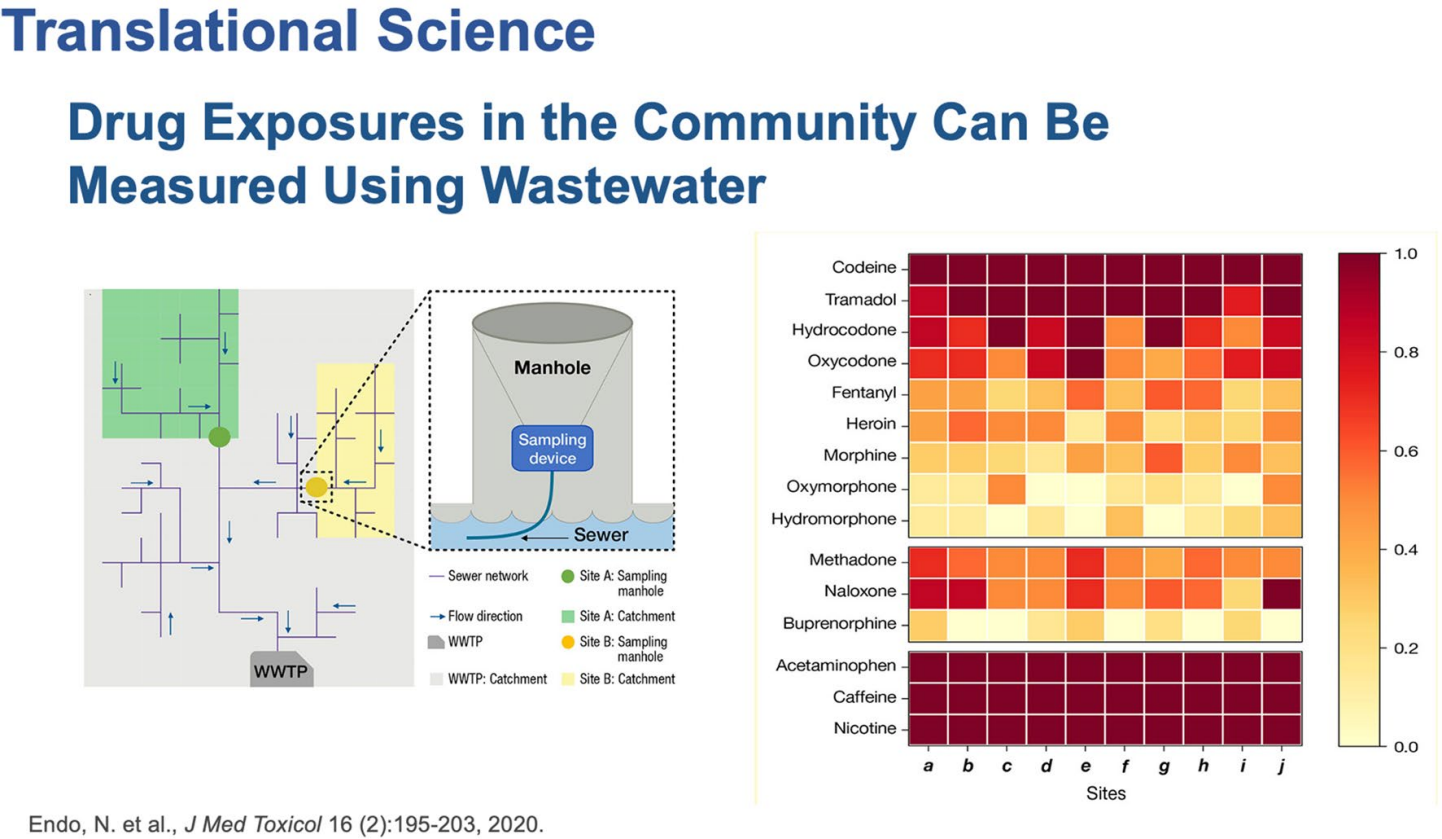
Using wastewater to assess drug exposure in the community

One of our initiatives for our translational program started before COVID to expand the use of wastewater testing for drugs in communities (Fig. 8) [8]. Because the technology has advanced, we can now measure extremely low concentrations of drugs in wastewater. It is relevant because they are new emerging drugs. Wastewater can be used to monitor xylazine and naloxone. It can determine if the people that are being prescribed naloxone are taking it. One of the states was saying they have been seeing xylazine from the coroner’s report and from the wastewater. There are also reports of contaminating the drug supply with Tylenol®. Based on the data, they are using it as a filler.

### Special Populations

**Fig. 9:**
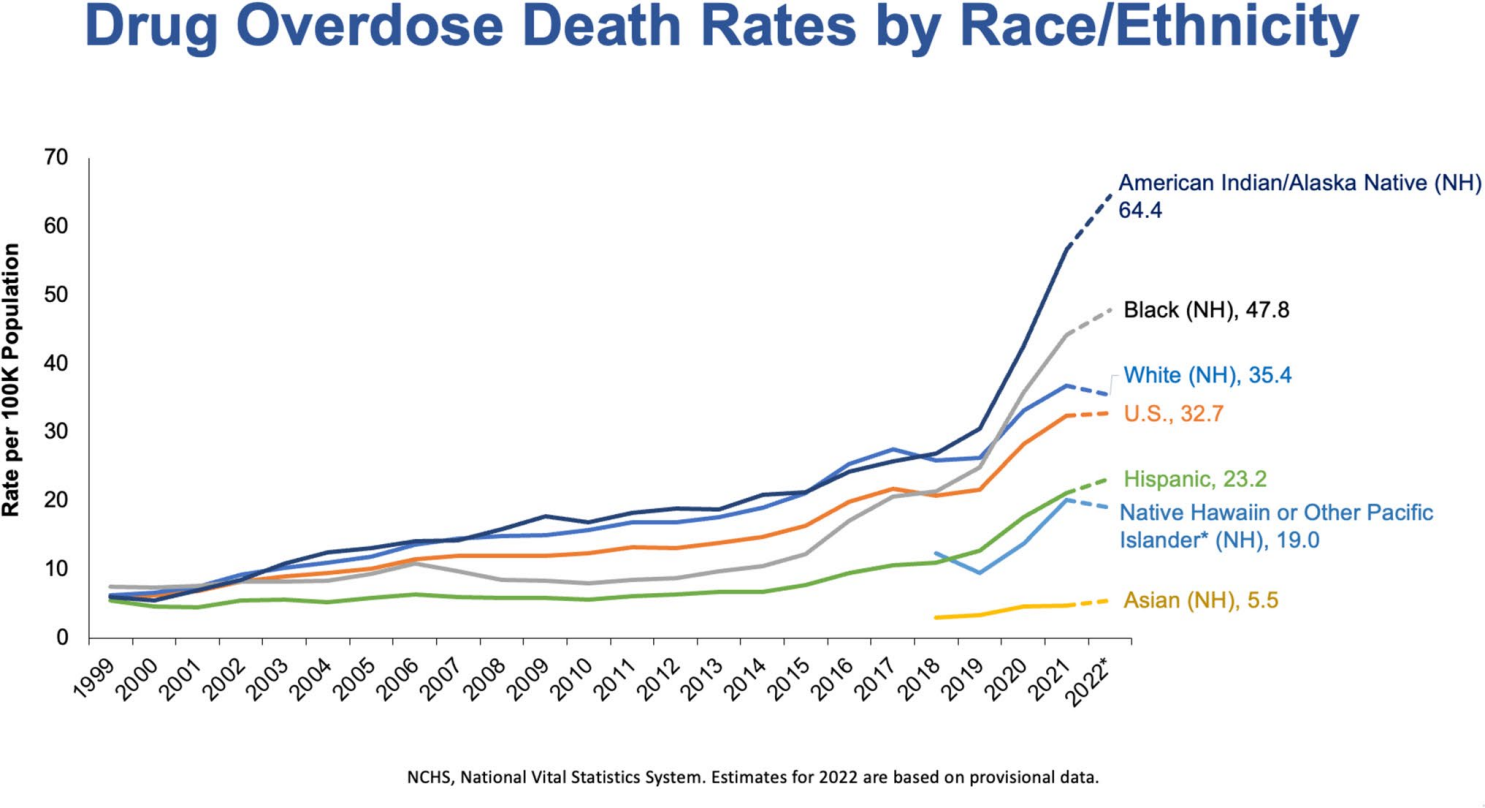
Drug Overdose rated by Race/Ethnicity

This is the rate of mortality of people per 100,000 in the United States by race/ethnicity (Fig. 9) [9]. Look at American Indians and Alaskan natives, their mortality rates are 65% per 100,000. Look at black Americans mortality rates, they are 50% higher than the rest of the United States. What is worrisome is not just the numbers themselves but that they have continued going up. If we were just paying attention to the overall average we could feel comfortable. Another group we are not controlling is teenagers. A group that we’re now seeing that have increases in mortality are people that are 65 or older. Neither teenagers nor people over 65 who are dying from fentanyl overdoses are seeking out fentanyl. They are seeking out other medications. Teenagers seek out psychotherapeutics, like medication to help prepare for an exam. Older people may be seeking medication because it may be easier to get and less expensive than getting it from providers. These are people we need to focus on (Fig. 9).

We have come to realize that if we are to address the overdose crisis, of course treatment is crucial. We need to mount much more proactive structures that enable us to intervene for prevention and sustained prevention so that we can protect people from overdoses. If we can have abstinence and then recovery that would be ideal, but in the meantime, people are dying. There are many things we can do to protect them so that we can eventually try to transition them into treatment.
